# Sphingosine Kinase Activity Is Not Required for Tumor Cell Viability

**DOI:** 10.1371/journal.pone.0068328

**Published:** 2013-07-05

**Authors:** Karen Rex, Shawn Jeffries, Matthew L. Brown, Timothy Carlson, Angela Coxon, Flordeliza Fajardo, Brendon Frank, Darin Gustin, Alexander Kamb, Paul D. Kassner, Shyun Li, Yihong Li, Kurt Morgenstern, Matthew Plant, Kim Quon, Astrid Ruefli-Brasse, Joanna Schmidt, Elissa Swearingen, Nigel Walker, Zhulun Wang, J. E. Vivienne Watson, Dineli Wickramasinghe, Mariwil Wong, Guifen Xu, Holger Wesche

**Affiliations:** 1 Oncology Research, Amgen Inc., Thousand Oaks, California, United States of America; 2 Medicinal Chemistry, Amgen Inc., South San Francisco, California, United States of America; 3 Pharmacokinetics & Drug Metabolism, Amgen Inc., South San Francisco, California, United States of America; 4 Discovery Sciences, Amgen Inc., South San Francisco, California, United States of America; 5 Genome Analysis Unit, Amgen Inc., South San Francisco, California, United States of America; 6 Molecular Structure and Characterization, Amgen Inc., Cambridge, Massachusetts, United States of America; 7 Therapeutic Innovation Unit, Amgen Inc., Seattle, Washington, United States of America; Ludwig-Maximilians University, Germany

## Abstract

Sphingosine kinases (SPHKs) are enzymes that phosphorylate the lipid sphingosine, leading to the formation of sphingosine-1-phosphate (S1P). In addition to the well established role of extracellular S1P as a mitogen and potent chemoattractant, SPHK activity has been postulated to be an important intracellular regulator of apoptosis. According to the proposed rheostat theory, SPHK activity shifts the intracellular balance from the pro-apoptotic sphingolipids ceramide and sphingosine to the mitogenic S1P, thereby determining the susceptibility of a cell to apoptotic stress. Despite numerous publications with supporting evidence, a clear experimental confirmation of the impact of this mechanism on tumor cell viability *in vitro* and *in vivo* has been hampered by the lack of suitable tool reagents. Utilizing a structure based design approach, we developed potent and specific SPHK1/2 inhibitors. These compounds completely inhibited intracellular S1P production in human cells and attenuated vascular permeability in mice, but did not lead to reduced tumor cell growth *in vitro* or *in vivo*. In addition, siRNA experiments targeting either SPHK1 or SPHK2 in a large panel of cell lines failed to demonstrate any statistically significant effects on cell viability. These results show that the SPHK rheostat does not play a major role in tumor cell viability, and that SPHKs might not be attractive targets for pharmacological intervention in the area of oncology.

## Introduction

Sphingosine-1-phosphate (S1P) is a pleiotropic phospholipid with many biological functions [1]. S1P is formed intracellularly through the phosphorylation of the sphingolipd sphingosine by sphingosine kinases (SPHKs). Mammals have two isoforms of SPHKs, SPHK1 and SPHK2. Some reports in the literature have suggested differential roles for these two isoforms [2], but knockout studies in mice suggest that they are at least partially redundant. Single knockouts of either isoform do not have a phenotype besides reduced plasma S1P levels, while the double knockout is embryonic lethal, highlighting the importance of this system during development, particular for neurogenesis and angiogenesis [3].

S1P is actively transported out of the cytosol, and can act through S1P receptors on a variety of target cells. Besides having mitogenic effects in several cellular systems [1], S1P plays an important role in lymphocyte trafficking. This role as a chemoattractant is primarily mediated through the S1P1 receptor and led to the development of the compound FTY720 (Fingolimid), which acts as a functional antagonist of S1P1 and is approved for the treatment of multiple sclerosis [4]. S1P can also act as a potent chemoattractant for endothelial cells, which is thought to contribute to its role in angiogenesis [5]. The importance of S1P1 for the sphingosine/S1P axis is further demonstrated by the S1P1 knock out phenotype, which phenocopies the double SPHK1/2 knockout [6]. This suggests that at least during embryogenesis, the biological effects of S1P are primarily mediated through this receptor.

In addition to the biological effects of S1P which are mediated through S1P receptors, a largely receptor-independent mechanism for sphingosine/S1P activity has been postulated, the rheostat theory [2], [7]. According to this theory, the response of cells to pro-apoptotic stimuli is thought to be determined in large part by the intracellular concentration of the interconvertible, pro-apoptotic lipids ceramide and sphingosine. SPHKs convert sphingosine to S1P, which is then either exported out of the cell (where it can act as a mitogen) or irreversibly cleaved by S1P lyase, leading to a decrease in the intracellular concentration of ceramide and sphingosine. Cells with high SPHK activity are thought to be more resistant to apoptosis, while reduced SPHK activity is thought to lead to increased sensitivity to apoptosis and decreased cell viability. It has been postulated that this sphingolipid rheostat might be an important contributor to tumor initiation and growth, making SPHKs an attractive target for pharmacological intervention in oncology [8].

Recently, studies suggesting a role of SPHK2 in the regulation of apoptosis have been published. It was shown that sphingolipids like ceramide can support BAX/BAK activation in biochemical assays, and that siRNAs targeting SPHK2 can increase apoptosis in cell lines [9]. While these studies confirm that sphingolipids can play a role in the process of apoptosis, and suggest a reasonable mechanism of action, the degree to which modulation of sphingolipid levels through inhibition of SPHK activity contributes to the regulation of apoptosis remains unclear.

Experimental proof for the rheostat theory has been hindered by the lack of suitable tools to conclusively study the effect of SPHK inhibition in cells and organisms. Initial studies were performed by the addition of sphingolipids and phospho-sphingolipids to tissue culture cell supernatants [7]. Since these lipids are very hydrophobic and accumulate in membranes and on hydrophobic surfaces, control of intracellular concentrations and exclusion of potential biological effects caused by membrane perturbations proved challenging. Subsequently, several SPHK inhibitors were described in the literature, e.g. SKII or SK1-I [10], [11], and studies with these compounds demonstrate that they are able to kill cells and in some cases increase the potency and/or efficacy of cytotoxic agents like platinum drugs [12]. These compounds are weak inhibitors of SPHK activity in biochemical assays. Some compounds have been shown to reduce intracellular S1P, but at concentrations that are much lower than their biochemical activity and that coincide with cytotoxic activity. More recently, potent and well characterized inhibitors such as PF-543 have been developed [13]. While the data obtained with PF-543 contradicts reports of the effect of SK1-I, and suggest that SK1-I’s biological activity is at least partially due to off target effects, it still does not conclusively address the rheostat theory, since both compounds do not inhibit SPHK2 significantly.

Here we report results obtained with representatives from a novel class of SPHK inhibitors, compound A and compound B. These compounds potently inhibit both human SPHK1 and SPHK2, and can reduce intracellular S1P levels below detectable levels. Nevertheless, complete inhibition of intracellular S1P production did not lead to any identifiable effects on cell viability in a panel of cell lines in either short term or long term assays. These findings are supported by experiments with SPHK1 or SPHK2 targeting siRNAs. In this setting, suppression of SPHK expression also failed to significantly affect cell viability. While SPHK inhibition in mice led to effects on vascular permeability in at least one mouse model, it did not affect tumor growth in the xenograft models tested. In summary, these results suggest that SPHK activity does not play an important role as a major regulator of apoptosis in tumor cells.

## Materials and Methods

### Determination of Biochemical SPHK Activity

A biochemical assay was used to measure the incorporation of ^33^P-radiolabeled phosphate from ATP into S1P. Reactions were performed in 96-well polystyrene high-binding microplates (Greiner) in a volume of 60 µl. Reaction mixtures contained 50 mM HEPES (pH 7.5), 3 mM MgCl2, 10 mM NaF, 1 µM ATP, 0.75 µCi of [γ-33P]ATP, 1.11 µM sphingosine (Biomol International) in the presence of either 0.005% Triton X-100 (SPHK1) or 0.0025% NP-40 (SPHK2). Enzymatic reactions were initiated by adding 30 nM of sphingosine kinase in the presence of 2 mM dithiothreitol (DTT). Reactions were terminated after 50 min at 30°C by adding 30 µL 7.5 M Guanidine HCl. Plates were then washed three times with 200 µl PBS before 50 ul of scintillate was added to each well. The assay was quantified by counting in a Packard TopCount (Perkin Elmer).

### Determination of Cellular C17 S1P Levels

All general biochemical reagents were obtained from Sigma-Aldrich (St. Louis, MO). DMSO was from Alfa Aesar (Ward Hill, MA). All 384-well microtitre plates used were obtained from Costar/Corning Inc. (Corning, NY). Skirted PCR plates were obtained from Phoenix Research Products (Candler, NC) and un-skirted PCR plates were obtained from BrandTech Scientific Inc (Essex, CT). High purity argon Gas was obtained from Airgas East (Salem, NH). Frozen cell pellets (1×10^6^ cells) were thawed and 75 µl of methanol was added. The samples were transferred to an un-skirted 96 well PCR plate after which they were mixed to a homogeneous solution. The plate was then sealed and pulsed for 6 seconds per well (E200-S0%250 mV500 setting) on a Covaris Adaptive Focused Acoustic (AFA) cell disruptor (Covaris, Woburn, MA). 130 µl of Dichloromethane was then added to each well after which plates were incubated at room temperature for 2 hours. Post incubation, 52 µl of ddH_2_O was added to induce phase separation. After the ddH_2_O addition, plates were spun in a Sorvall (Thermo Scientific, Waltham, MA) RC-4 centrifuge (2000 rpm for 5 minutes) to achieve full phase separation. 70 µl of the upper phase was then transferred to a new plate and supplemented with 70 µl of (80∶20) Methanol:ddH_2_O, 2% Formic Acid and 150 mM Ammonium Acetate. 45 µl of this final sample was transferred to a 384 well plate and analyzed on a RapidfireTM (Biocius, Wakefield, MA) ultra-high throughput chromatography system interfaced with a TSQ Vantage triple stage quadrupole mass spectrometer (Thermo Scientific, Waltham, MA). For RapidfireTM analysis, 20 µl of the assay sample was aspirated (250 ms) from each well of a 384-well reaction microtiter plate. The sample (10 µl) was then injected onto a proprietary C18 solid-phase extraction cartridge under conditions designed to bind the analyte of interest. After an aqueous wash ((80∶20) Methanol/ddH_2_O containing 75 mM NH_4_OH and 0.1% formic acid) to remove the S1P extraction components that interfere with mass spectrometry, C17-S1P was eluted ((99∶1) Methanol/ddH2O containing 0.1% formic acid) and analyzed by inline triple stage quadrupole mass spectrometry. The RapidFireTM sipper was washed between sample injections to minimize sample carryover using an organic (200 ms) and aqueous (500 ms) solvent. The total sampling time, including sipper washes and data acquisition was 6.2 seconds per sample or 39.7 minutes per 384-well microtitre plate. All Mass Spectrometry was performed with a ThermoFisher TSQ Vantage triple stage quadrupole using an electrospray HESI-II Probe in the negative ion mode (Thermo Scientific, Waltham, MA). C17-S1P was monitored using a selective reaction monitoring protocol which detected the loss of a phosphate molecule from the parent ion C17-S1P. The parent ion mass in Q1 was 364.35 amu and the corresponding daughter ion in Q3 was 79.04 amu. Optimal instrument settings for the TSQ Vantage were as follows: sheath gas pressure = 30 psi, auxiliary gas pressure = 25 psi, declustering voltage = 0 V, spray voltage = 4000 V, vaporizer temperature = 387°C, argon collision pressure = 2.2 mTorr, capillary temperature = 398°C, dwell time = 0.1 seconds, collision energy = 42 V and S-lens amplitude = 87 V. The area under the daughter ion peaks (AUC) were quantified using RapidfireTM integrator software.

### Viability Assays

Cell lines were obtained from ATCC and cultured in Dulbecco’s Modification of Eagle’s Medium (Corning® cellgro 10-013) supplemented with 5% FBS and 1% P/S or in Minimum Essential Medium (Corning® cellgro® 10-010) supplemented with 10% FBS, 0.1 mM MEM Nonessential Amino Acids (Corning® cellgro 25-025), 1.0 mM sodium pyruvate (Corning® cellgro 25-000) and 1% P/S. Cells were seeded at a density of 5000 cells per well in 96-well cell culture plates on day1. Media was changed with or without serum and dosed with compound on Day 2. On day5 the Cell Viability assay was performed with CellTiter-Glo® Reagent (Promega) according to the manufacturers instruction.

### Colony Formation Assays

LN-229 cells were cultured in Dulbecco’s Modification of Eagle’s Medium (Corning® cellgro 10-013) supplement with 5%FBS and 1% P/S. On Day 1 cells were seeded on 6-well tissue culture plates at density of 800 cells per well. On Day 2 compounds were added and cells were incubated for 15 days at 37°C. Cells were then stained with Crystal violet.

### Determination of Compound and S1P Plasma Concentrations

For S1P measurements, plasma was extracted with 0.1% formic acid/methanol containing an internal standard (C17-S1P) and subjected to LC-MS/MS analysis. Chromatographic separation was achieved with a Kinetix column (Phenomenex), and quantitation was performed on a API4000 triple quadrupole mass spectrometer (AB Sciex, Foster City, CA) with an electrospray interface using multiple reaction monitoring (MRM) in the negative ionization mode. The method provided linear response over a concentration range of 1 to 2000 ng/ml. A separate aliquot of plasma was extracted with acetonitrile containing an internal standard (structural analog) and subjected to reversed-phase liquid chromatographic using a Shiseido UG Capcell C18, 50×2 mm analytical column (Phenomenex). MS/MS analysis was conducted on a API4000 with an electrospray interface using multiple reaction monitoring (MRM) in the positive ionization mode.

#### Animals for *in vivo* studies

All *in vivo* studies were conducted in accordance with the guidelines of the Amgen Animal Care and Use Committee, which approved this study. Female athymic nude mice and C57Bl/6 mice aged 6–8 weeks were obtained from Harlan Sprague Dawley Inc. The facilities where experiments involving animals were conducted were approved by the Association for Assessment and Acreditation of Laboratory Animal Care.

#### Pharmacokinetic/pharmacodynamic studies

Female athymic nude mice were assigned to one of fifteen treatment groups. Compound A was administered by oral gavage at doses of 10, 30, 100, 300 mg/kg or vehicle. At various times after dosing (2 to 24 h), mice were sacrificed and plasma collected to determine S1P levels and compound concentrations. Data are mean ± SE (n = 5). P values correspond to statistical difference between groups treated with vehicle and compound A as determined by one-way analysis of variance (ANOVA) followed by Dunnett post hoc testing using JMP software (version 8.0.2: SAS Institute, Inc., Cary NC). S1P and drug concentration were determined by LC-MS/MS.

#### Vascular permeability assays

Vascular permeability was induced using a modified Miles assay [14], [15]. Twenty-four hours after implantation of cells, mice were treated with Vehicle, the VEGFR2 inhibitor motesanib or compound A for various periods of time followed by injection of 0.1 ml of 1% Evans blue dye. Data represent mean +/− SE (n = 4–5). Statistical analysis was done with one-way ANOVA using JMP 8.0.2 software (SAS Inc.). Dunnett’s post hoc test was used to determine p values.

#### Tumor xenograft models

MDA-MB-231 cells were purchased from the American Type Culture Collection (ATCC) and maintained in DMEM high glucose with 10% fetal bovine serum (FBS) and 1x-L-glutamine. Mice were injected subcutaneously with 5×10^6^ cells in 30% Matrigel (BD Biosciences, San Jose, CA). Eighteen days later, when tumors were approximately 200 mm^3^, mice were randomized and treated with either vehicle, compound A or Docetaxel. Vehicle and compound A were administered by oral gavage daily. Taxotere was administered by intraperitoneal injection once a week. Tumor dimensions were assessed twice weekly with a Pro-Max electronic digital caliper (Sylvac, Crissier, Switzerland) and tumor volume was calculated using the formula: length x width x height and expressed as mm^3^. Data are expressed as mean +/− SE (n = 7–10). Repeated-measures analysis of variance (RMANOVA) followed by Dunnett’s post hoc test for multiple comparisons was used to evaluate statistical significance of observed differences. Body weight was recorded twice weekly as an index of toxicity.

#### High throughput siRNA screens

siRNAs from Qiagen Inc. (Valencia, CA) or from Thermo Scientific (Dharmacon Products, Lafayette CO) were used to create libraries with 4–20 siRNAs for each gene. Each siRNA was individually transfected into cells using Lipofectamine RNAiMAX transfection reagent (Life Technologies, Carlsbad CA). siRNAs from a library plate were diluted in serum-free media to a volume of 6 µl. Transfection reagents diluted in serum-free media to a volume of 5 µl were added to each well using a BiomekFx Robot (Beckman Coulter). After a 20-minute room temperature incubation, cells were added to the plates using a Multidrop (ThermoScientific). After 96 or 120 hours, cell viability was determined with CellTiterGlo™ (Promega, Madison, WI) and luminescence was measured on a luminometer according to the manufacturer’s instructions. The final siRNA concentrations (10–30 nM) and RNAiMAX volume used per well (0.02–0.1 µl) and plating cell density (500–1500 cells/well) varied by cell line. Most cell lines were screened using multiple transfection conditions. Results from the viability assays were processed through Screener® (Genedata, Basel Switzerland). The effect of knocking down a given gene on viability was summarized as a p value, by combining the results of all of the siRNAs targeting that gene using the inverse normal method of Stouffer [16], modified as described in [17]. p-values were corrected for multiple hypothesis testing using the method of Benjamini and Hochberg [18].

## Results

### Development of Sphingosine Kinase Inhibitors

**Figure 1:**
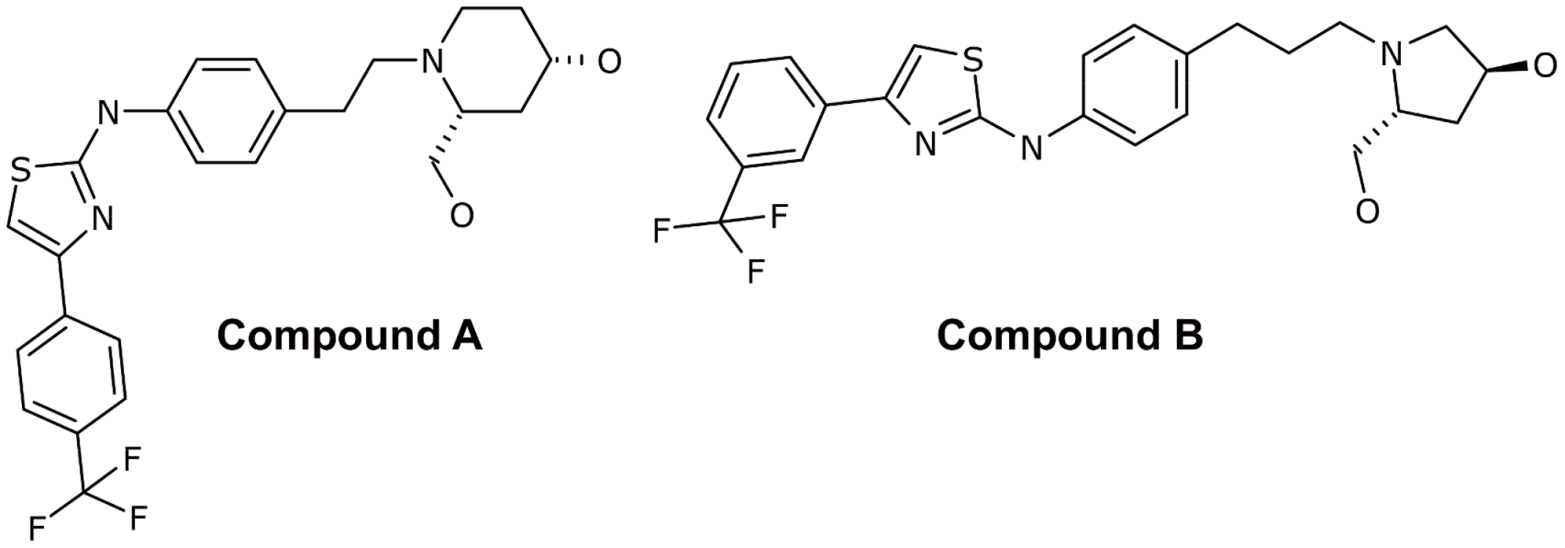
Structure of Compounds A and B.

Based on the crystal structure of human SPHK1 [19], potent and specific pan SPHK inhibitors were developed in a structure guided design approach (Darin Gustin, manuscript submitted). Two of these inhibitors, compound A and compound B, were utilized to explore the effects of SPHK inhibition on tumor cell viability (figure 1). Both compounds are potent, sphingosine competitive inhibitors of human SPHK1 and SPHK2. They also inhibit murine SPHK1, but not murine SPHK2 (table 1). This structural class of compounds does not significantly inhibit 100 protein kinases tested at a concentration of 1 µM in biochemical assays (data not shown), and has no agonistic or antagonistic activity on S1P receptors (data not shown).

**Table 1:** Inhibition of human and murine SPHK activity in biochemical assays.

	Compound A	Compound B
IC_50_ (hSPHK1) [µM]	0.020	0.008
IC_50_ (hSPHK2) [µM]	0.114	0.060
IC_50_ (mSPHK1) [µM]	0.064	0.055
IC_50_ (mSPHK2) [µM]	>5	>5

### Inhibition of Cellular SPHK Activity

**Figure 2:**
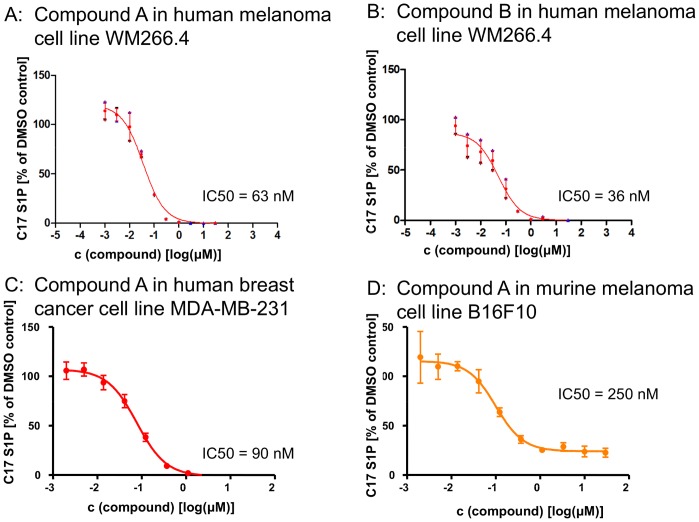
Inhibition of cellular SPHK activity in tumor cell lines. Two hours after addition of C17 sphingosine, cells were lysed and levels of C17 S1P were determined.

The cellular activity of compounds A and B was determined by monitoring intracellular sphingosine-1-phosphate levels with mass spectroscopy. Following complete inhibition of SPHK activity, endogenous S1P levels decreased over 24 h, demonstrating the depletion of intracellular S1P pools. The rate of the decrease in S1P levels is determined by both inhibition of S1P production and degradation of S1P. Interpretation of results is complicated by potential biological consequences of S1P depletion, e.g. the potential loss of viability, and the impact of feedback loops. In order to assess inhibition of sphingosine kinase activity more directly, an assay with exogenous C17 sphingosine was developed. Naturally occurring sphingolipids have an even number of carbon atoms in their lipid backbone, with C18 sphingosine being the most prominent form. Exogenous C17 sphingosine is rapidly taken up by cell lines and converted to C17 sphingosine-1-phosphate. Measurements of C17 S1P levels 2 h after addition of C17 sphingosine to tissue culture cells established a robust and reproducible means to assess cellular SPHK activity [20]. Compounds A and B inhibited SPHK activity in human cell lines with IC_50_s in the double digit nanomolar range, and reached complete inhibition at concentrations between 0.3 and 1 µM. In murine cell lines, the compounds were less potent and less efficacious, consistent with a lack of inhibition of murine SPHK2 (figure 2).

### Effects of SPHK Inhibition on Cell Viability

**Figure 3:**
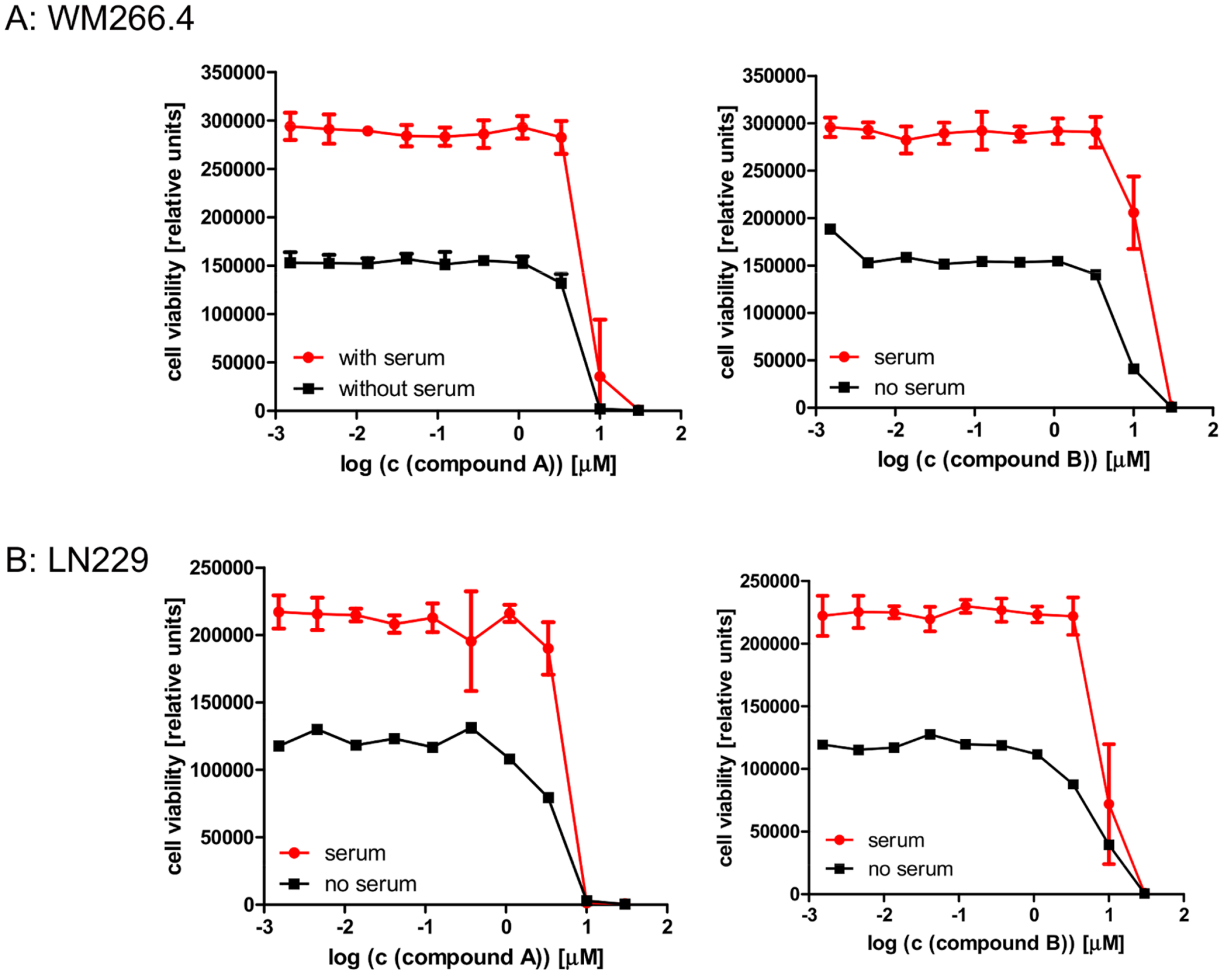
Effects of SPHK inhibition on cell viability. The human melanoma cell line WM266.4 (panel A) and the human glioblastoma cell line LN229 (panel B) were treated for 72 h with the indicated concentrations of compound A (left panel) and compound B (right panel). Viability was assessed after 72 h.

**Figure 4:**
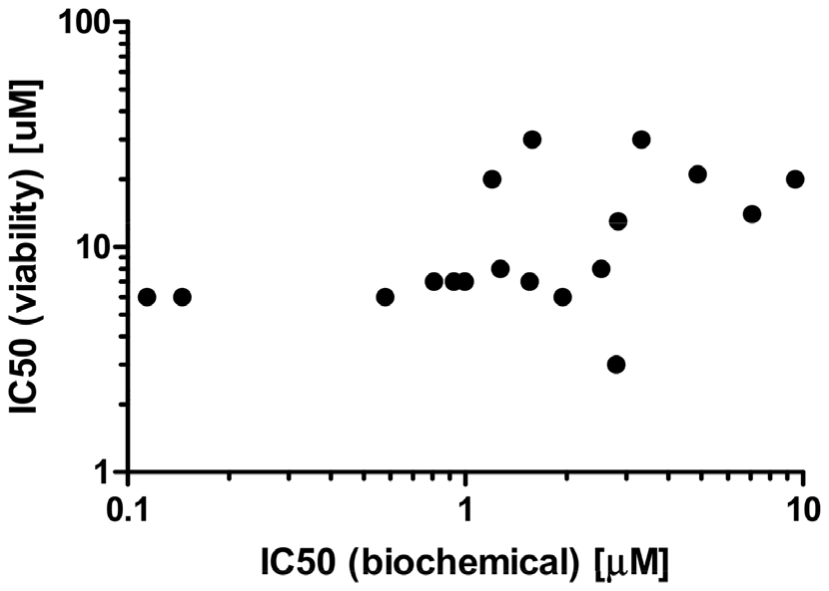
Correlation between SPHK inhibition and cell viability. A panel of 18 compounds structurally related to compounds A and B was tested in biochemical hSPHK1 assays (inflection point IC_50_s plotted on x-axis) and 72 h viability assays in WM266.4 cells (inflection point IC_50_s plotted on y-axis).

S1P has been reported to be important for tumor cell viability, and reduction of intracellular S1P levels has been associated with greater cellular sensitivity to pro-apoptotic stress [2], [7]. Compounds A and B were used to test this hypothesis experimentally. A panel of cell lines likely to be sensitive to SPHK inhibition was assembled based on literature reports, internal siRNA data and SPHK expression levels [5], [9], [21]. Viability was monitored after 72 h of SPHK inhibition under regular culture conditions to assess direct effects on cell viability, and under serum starvation to assess the effects of SPHK inhibition under stress conditions. Complete inhibition of SPHK activity at concentrations of 1 µM compound did not result in reduced viability under the conditions studied (see figure 3 for representative results and table 2 for a summary of the data). Cell death was only observed at higher concentrations (3 to 30 µM). This cell death is likely caused by the physicochemical properties of the compounds. Like all substrate competitive SPHK inhibitors, these compounds have a polar headgroup and a lipophilic tail, and can act as detergents at higher concentrations. Time lapse microscopy revealed that cell death occurs immediately after compound addition (data not shown) and is likely caused by cell lysis as opposed to SPHK inhibition. This assumption is supported by an analysis of 18 SPHK inhibitors structurally related to compounds A and B. These compounds have similar physicochemical properties, yet differ in their ability to inhibit SPHKs with biochemical IC_50_s between 0.1 and 1 µM. The degree of SPHK inhibition does not correlate with the effect on WM266.4 cell viability (figure 4).

**Table 2:** Effects of SPHK inhibition on cell viability in 72 h assays (the reported IC_50_ values are the inflection points of the titration curves).

Number	Cell line	Tissue type	Compound A IC_50_ (µM)	Compound B IC_50_ (µM)
1	Colo 320	Colon	5.1	9.4
2	LN18	Brain	4.1	5.5
3	LN229	Brain	4.8	8.7
4	HA-1	Kidney	6.3	15.5
5	HS294T	Skin	6.0	10.1
6	UACC257	Skin	4.4	6.5
7	Saos2	Bone	7.0	13.5
8	A375	Skin	8.3	17.4
9	HeLa	Cervix	6.4	14.3
10	LOX	Skin	4.0	6.2
11	WM266-4	Skin	6.0	13.2

**Figure 5:**
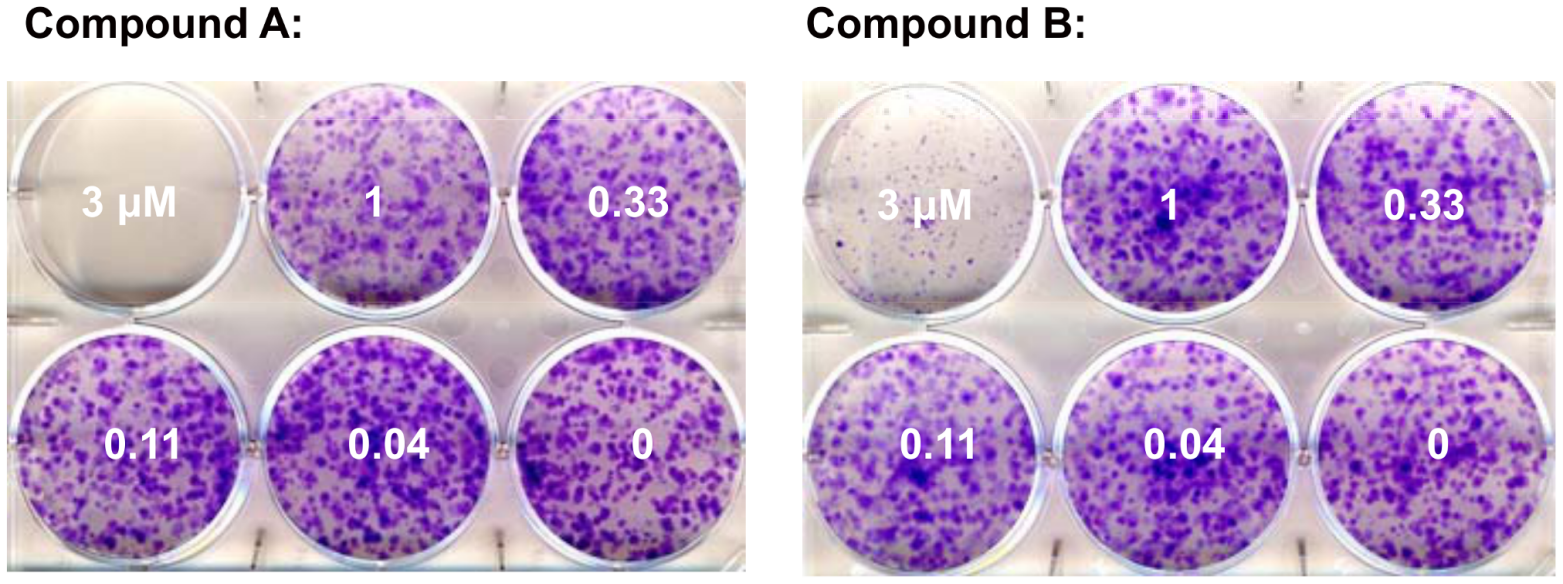
Effects of SPHK inhibition in colony formation assays. LN229 cells were seeded at a density of 800 cells/well in a six well plate in the presence of the indicated concentrations of SPHK inhibitor. After 15 days, cells were fixed and stained with crystal violet.

The long term effect of SPHK inhibition on cell viability was studied in the glioblastoma cell line LN229. Consistent with the results from the 72 h assays, 15 day colony formation assays did not reveal any impact of SPHK inhibition on colony formation and colony growth with up to 1 µM of compound. Concentrations of more than 3 µM led to immediate cell death due to cell lysis and prevented the formation of colonies (figure 5).

### Evaluation of Compound SKII

**Figure 6:**
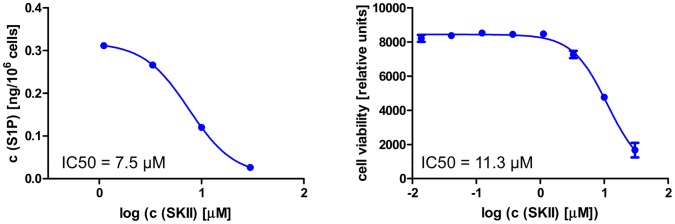
Cellular activity of literature compound SKII. The left panels shows levels of endogenous S1P 24 hours after compound treatment, the right panel depicts the result of 72 h viability assays performed in parallel in the human melanoma cell line LOX. The IC_50_s reflect the inflection point of the titration curve.

Several SPHK inhibitors have been reported in the literature, and have been utilized to to support the rheostat theory [2]. While some of these compounds inhibit only one isoform of SPHK or do not show any activity in biochemical assays, a compound termed SKII has clearly detectable activity against both SPHK1 and SPHK2 [10]. The biochemical activity against human SPHKs is between 10 and 20 µM, while activity against the murine enzymes is too low to be detected. Treatment of the melanoma cell line LOX for 24 h led to reductions of endogenous S1P levels with an IC_50_ of 7.5 µM. SKII treatment led to cell death in the same cell line with an IC_50_ of 13.5 µM within 72 h (figure 6 and table 3). Since complete inhibition of S1P production with compounds A or B does not have an effect on cell viability, it appears likely that at least some of SKII’s biological effects are off target and independent of sphingosine kinases.

**Table 3:** Biochemical and cellular activity of SKII.

	IC_50_ of SKII
hSPHK1 (biochemical) [µM]	17.5
hSPHK2 (biochemical) [µM]	13.0
mSPHK1 (biochemical) [µM]	>10
mSPHK2 (biochemical) [µM]	>10
Endogenous S1P production (LOX) [µM]	7.5
Viability (LOX cells) [µM]	11.3

### Activity of Compound A *in vivo*

**Figure 7:**
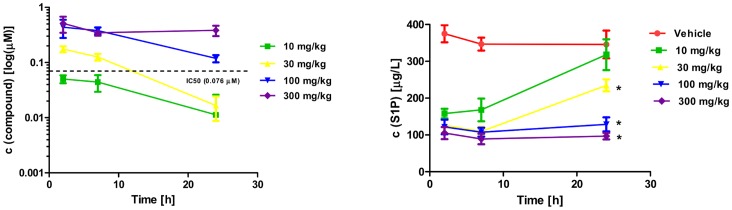
Phamacokinetic and pharmacodynamic properties of compound A *in vivo*. Compound A was administered to athymic nude mice by oral gavage. At the indicated time points, blood was collected and plasma levels of compound A (left panel) as well as S1P (right panel) were determined. Compound levels were corrected for binding to murine plasma, and concentrations of free compound A is depicted in this graph. *P<0.05 compared to vehicle.

The inhibition of sphingosine kinase was evaluated in a pharmacodynamic assay. Compound A was administered by oral gavage in mice (10, 30, 100 and 300 mg/kg). At 2, 7 and 24 hours post dose, plasma was collected and levels of S1P and plasma drug concentration were determined. Compound A is orally bioavailable, and a single dose of 100 to 300 mg/kg maintains a plasma concentration of free compound A that is above the *in vitro* IC_50_ for SPHK inhibition for more than 24 hours (figure 7, left panel). The concentration of free compound A was calculated from the total compound A plasma concentration based on the experimentally determined percentage of compound bound to murine plasma. The inhibition of SPHK activity led to a dose dependent reduction in plasma levels of S1P, with a maximum inhibition of 72% at a corresponding plasma exposure of 0.38 µM (figure 7, right panel). This result is consistent with the observation in cell lines (figure 2D). Compound A treatment leads to complete inhibition of SPHK activity and S1P production in human cell lines, but in murine cell lines as well as in mice, the lack of mSPHK2 inhibition is reflected in 20 to 30% remaining SPHK activity.

**Figure 8:**
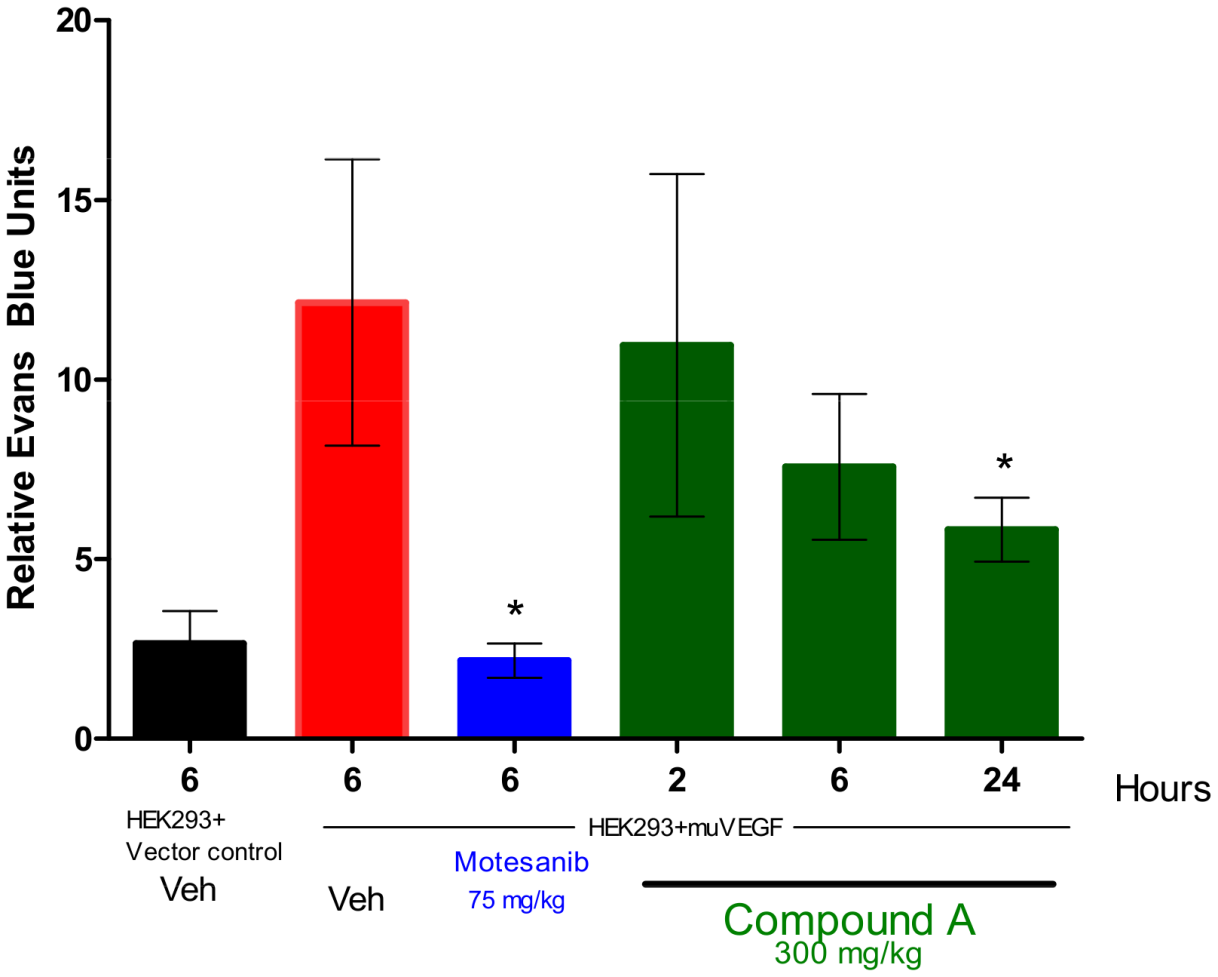
Inhibition of VEGF-induced vascular permeability in mice treated with Compound A. HEK 293 cells transfected with murine VEGF or vector were mixed with Matrigel and injected s.c. into female C57Bl/6 mice. A single dose of compound A was given by oral gavage 24 hours after implantation of cells. At various time points after administration, vascular permeability in the skin overlying the Matrigel plug was measured by quantifying the extravasation of Evans blue dye. Columns, relative Evans blue units (n = 5) per group; bars, SE *, P<0.0001, significant difference from VEGF plus vehicle-injected control mice.

A prominent role of S1P in murine angiogenesis has been established by the SPHK1/2 double knockout [6]. Vascular permeability assays were performed to see if SPHK inhibition affects this process. For this assay, 293 cells overexpressing murine VEGF or vector control were injected s.c. into mice. VEGF mediated effects on vascular permeability were measured by determining the extravasation of Evans blue dye into skin. Compound A at a dose of 300 mg/kg can induce a time-dependent decrease in vascular permeability with maximum inhibition at 24 hours (P<0.0001) (figure 8), demonstrating biologic activity of SPHK inhibition in mice. Compared to the positive control, the VEGFR2 inhibitor motesanib, the effects are delayed, of lower magnitude and appear to be mouse strain specific. An identical experiment in athymic nude mice did not lead to significant effects on vascular permeability (data not shown).

**Figure 9:**
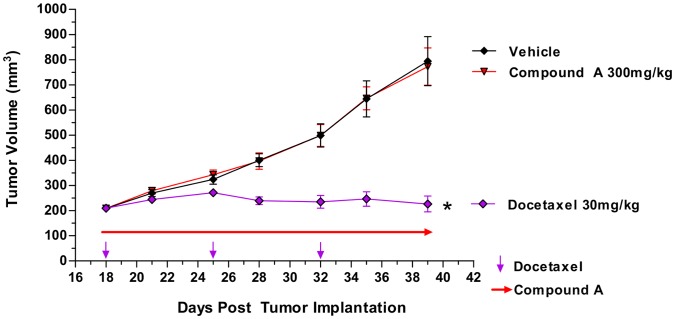
Effect of Compound A on the growth of MDA-MB-231 xenograft tumors. Female athymic nude mice were injected with 5×10^6^ MDA-MB-231 cells on day 0. Treatment with compound A (300 mg/kg/dose QD) or Taxotere (30 mg/kg, once per week) was initiated on day 18 when tumors reached ∼ 200 mm^3^. (n = 7–10 per group); bars, SE. *, P<0.0001, compared to vehicle.

Since compound A does not inhibit murine SPHK2, the human MDA-MB-231 xenograft model was utilized to study the effect of complete SPHK inhibition on tumor growth *in vivo*. Here, athymic nude mice were implanted s.c. with human MDA-MD-231 breast cancer cells. Dosing was initiated 18 days later when the tumors had reached ∼ 200 mm^3^. Compound A (300 mg/kg daily) was administered by oral gavage, the positive control Docetaxel (30 mg/kg) was administered by intraperitoneal injection once per week. In this particular study, Compound A was not tolerated in 3 out of the 10 mice. The cause of death was unknown; however neither death nor body weight loss were observed in other *in vivo* studies. The exposure achieved with this dose of compound A completely inhibited MDA-MB-231 S1P production *in vitro* (figure 2) and reduced host S1P levels by more than 70% (figure 7). This reduction in S1P levels did not lead to significant inhibition of tumor growth *in vivo* (figure 9).

### siRNA Knockown of SPHKs *in vitro*

**Figure 10:**
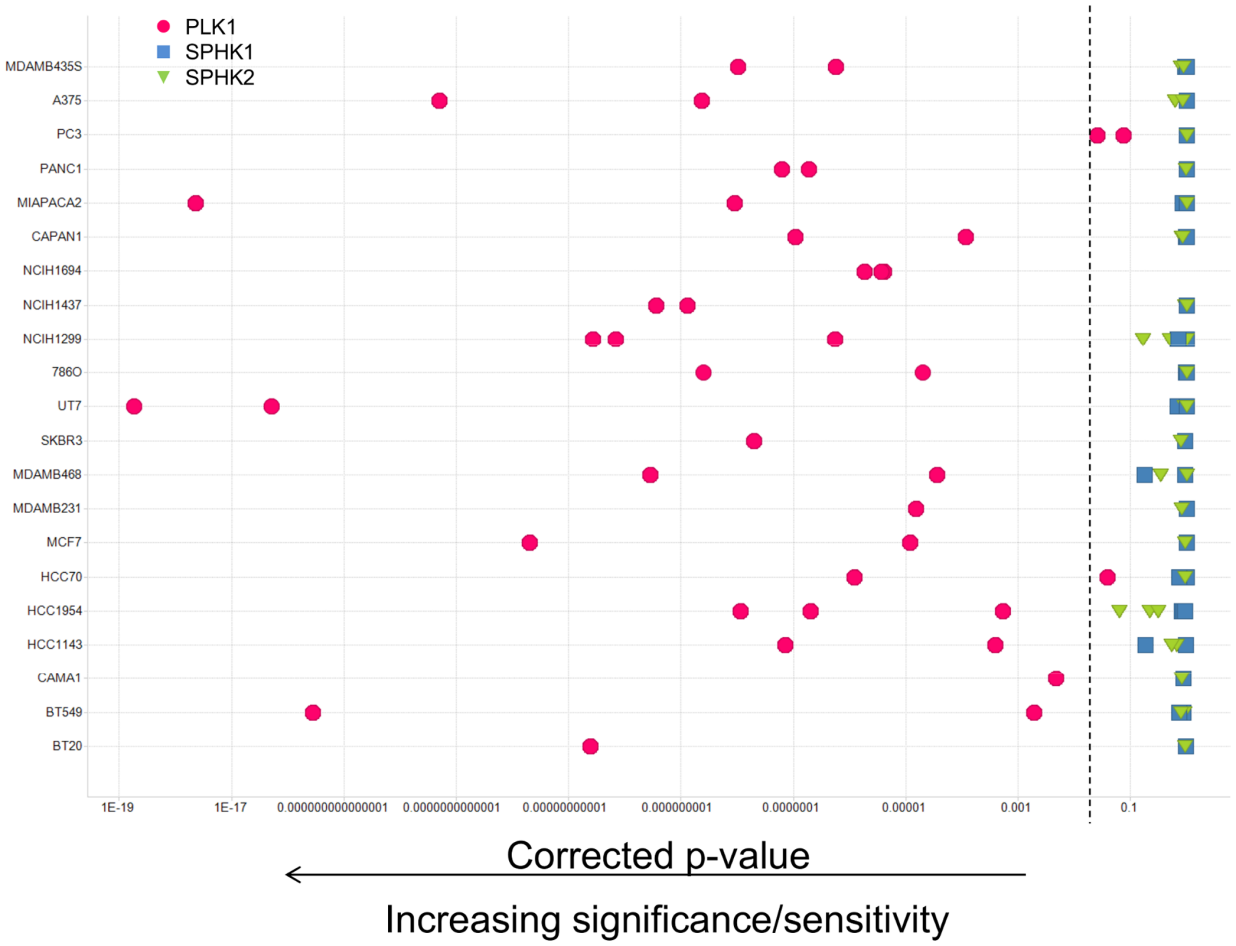
Reduction of SPHK expression by RNAi in a panel of cancer cell lines. Multiple cancer cell lines were transfected in a high-throughput format with libraries of siRNA containing multiple triggers for each gene, and cell viability was determined 96 or 120 hours after transfection. The statistical significance of the observed effects was calculated (see materials and methods) and expressed as a p value. Polo like kinase 1 (PLK1) served as a positive control. Each symbol represents the result of one siRNA screen. Most cell lines were tested several times using different transfection conditions. Symbols to the left of the dashed line (p<0.05) indicate a statistically significant effect of gene knockdown on cell viability in a given experiment.

**Figure 11:**
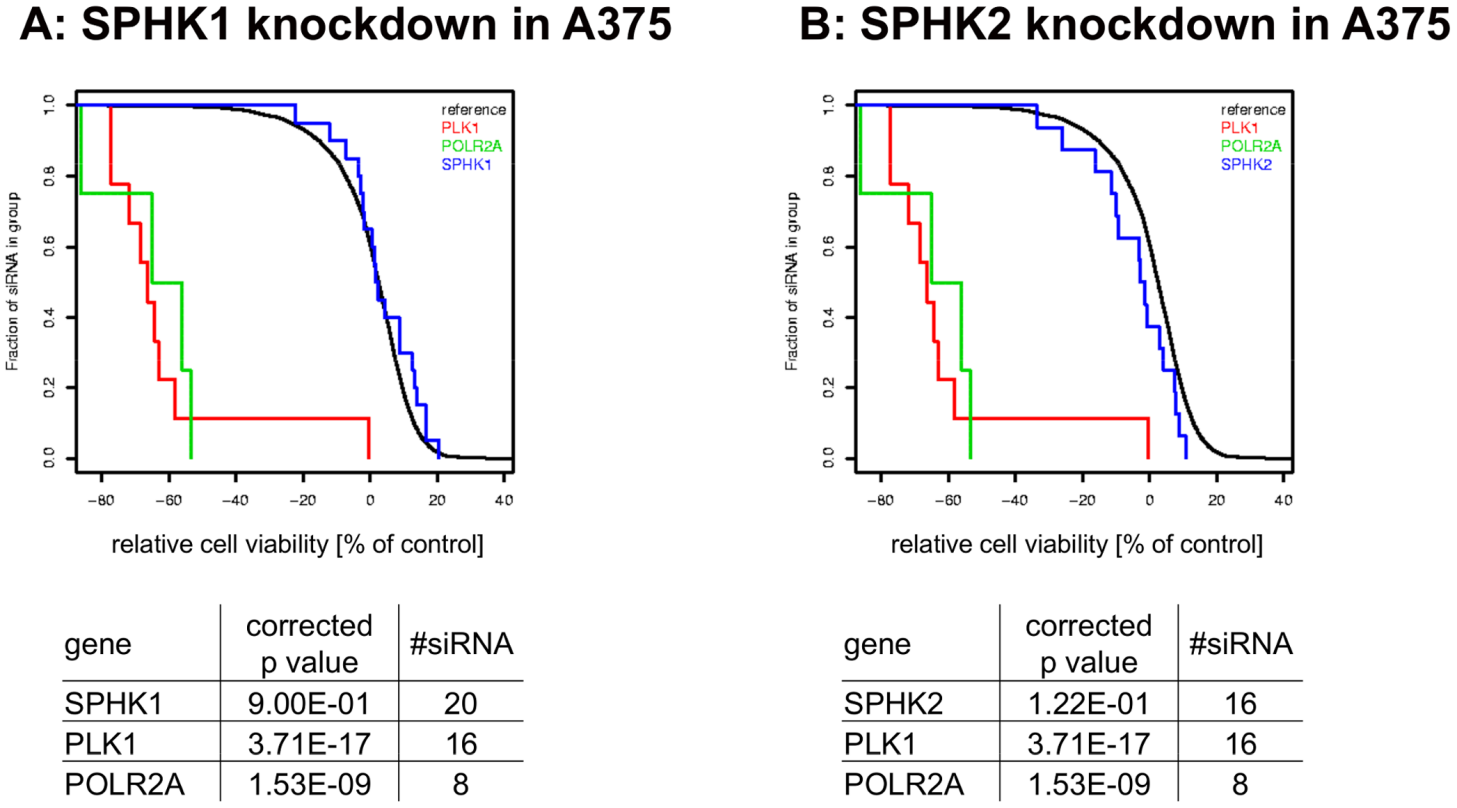
siRNA experiments in A375 cells. SPHK1 (panel A) and SPHK2 (panel B) as well as the cytotoxic controls PLK1 and POLR2A were targeted with numerous siRNAs in the melanoma cell line A375. Each vertical line represents the effects of an individual siRNA transfection on relative cell viability, with negative values representing cell killing. Statistical significance was calculated as described in Materials and Methods.

SPHK mediated effects on tumor cell viability that are independent of SPHK’s kinase activity were assessed in siRNA experiments. Since the interpretation of individual siRNA experiments with a viability readout can be complicated by off-target effects of the siRNAs and incomplete knockdown of the target, a high throughput approach was pursued to enable a statistical analysis of the results at the gene level. More than 80 tumor cell lines were transfected with libraries of individual siRNAs with a high degree of redundancy, including 20 siRNAs targeting each SPHK isoform. Each siRNA’s effect on cell viability was determined after 96 to 120 h and p values were calculated to determine if knocking down specific genes significantly affected cell viability (see Materials and Methods for details). In contrast to the positive control polo like kinase (PLK1), knockdown of either SPHK1 or SPHK2 did not result in significant reductions in viability in any of the cell lines tested (figure 10 is showing representative results for 20 cell lines). Figure 11 depicts the results for the melanoma cell line A375 in detail. The 20 siRNAs targeting SPHK1 (panel A) or SPHK2 (panel B) are indistinguishable from the reference siRNAs, while toxic genes like PLK1 or POLR2A have highly significant effects on cell viability.

## Discussion

Sphingolipids play a role in many important biological processes [4], [7]. Sphingosine-1-phosphate and its receptors play a significant role in lymphocyte migration, and blocking S1P function with FTY720 (Fingolimod) has been clinically shown to modify the course of inflammatory diseases [21]. Mice deficient for S1P1 or both sphingosine kinases, which are required for the production of S1P from sphingosine, show severe defects in the development of the nervous system and in angiogenesis [6]. The role of S1P in tumor angiogenesis has been further demonstrated in experiments with blocking monoclonal antibodies, which lead to significant reductions in tumor growth in several xenograft models [22]. In addition to the proliferative and migratory effects of secreted S1P, the rheostat theory postulates that the intracellular balance between S1P and sphingosine is important for cell viability and the cellular response to pro-apoptotic stimuli [7]. Sphingosine kinase activity can shift the intracellular sphingolipid pool from the pro-apoptotic lipids ceramide and sphingosine to the mitogenic phospholipid S1P, which then can be exported out of the cell, inducing a further depletion of the intracellular pro apoptotic lipid pool. According to this theory, inhibition of sphingosine kinases will lead to an accumulation of ceramide and sphingosine, resulting in apoptosis and reduced cell viability. The validation of this theory has been hampered by the lack of suitable tool compounds. Nevertheless, a number of reports show supporting evidence obtained with the available, imperfect tools (exogenously added lipids, partial SPHK knockdown with siRNA, SPHK inhibitors) [2], [3], [12]. The reported SPHK inhibitors are either not potent enough, or do not inhibit both sphingosine kinases sufficiently to allow firm conclusions. One example is the compound SKII, which has been widely used throughout the literature [5], [10]. Despite being one of the best characterized inhibitors, it is only moderately potent. As shown in figure 6, it induces cell death at concentrations similar to those at which it affects intracellular S1P levels. More potent compounds like compound A and compound B can reduce S1P levels below quantifiable limits without reducing viability in the same cell line, suggesting that the biological effects observed with SKII are not solely caused by SPHK inhibition (figure 2, 3 and table 2). A recent report performed with the potent and specific SPHK1 inhibitor PF-543 failed to demonstrate an effect of SPHK1 inhibition on tumor cell viability, although the role of SPHK2 could not be addressed, since these inhibitors are specific for SPHK1 [21].

Compounds A and B on the contrary, which were obtained through a structure guided design effort based on the crystal structure of sphingosine bound to human SPHK1 (Zhulun Wang and Darin Gustin, manuscripts submitted), inhibit both isoforms of human SPHK similarly. They are potent and reduce the concentration of cellular S1P with IC_50_s in the double digit nanomolar range in all human cell lines tested (table 1, figure 2). At concentrations below one micromolar, both compounds completely suppress the formation of S1P in tumor cell lines. At the same concentration, these compounds do not affect viability in a panel of 10 tumor cell lines in 72 hour proliferation assays (figure 3, table 3). In order to mimic cellular stress, the assay was carried out in the absence and presence of calf serum. To rule out that the inhibition of SPHKs can only be detected in longer term assays, we performed colony formation assays in the glioblastoma cell line LN229. LN229 has been previously reported to be sensitive to the SPHK1 inhibitor SK1-I [6]. When treated with compounds A or B at concentrations up to 1 µM, viability was unchanged in 72 h proliferation and 15 day colony formation assays, even though the production of S1P was completely inhibited (table 3, figure 5). The results reported with SK1-I and those obtained with compounds A and B appear contradictory, but it has to be considered that SK1-I is reported to be a very weak and SPHK1 specific inhibitor with a biochemical IC_50_ in the micromolar range [13]. In contrast to compounds A and B, SK1-I affects cellular S1P levels only moderately and at concentrations that coincide with reduced viability. This suggests that SPHKs are not the only target of SK1-I, and confounds the interpretation of the published results.

While compound A and compound B inhibit SPHK activity without affecting viability, it has to be noted that both compounds kill cells at higher concentrations. The IC_50_s in viability assays are two orders of magnitude higher than the IC_50_ obtained for cellular S1P production (figure 2 and figures 3). One likely explanation of the cellular toxicity is their physicochemical properties. Both molecules have polar head groups and lipophilic tails, and in analogy to phospholipids are likely able to disrupt plasma membranes at high concentrations, similar to a detergent. The observed cell death at high concentrations occurs immediately after compound addition, consistent with cell lysis. In addition, an analysis of 18 structurally related analogs with similar physicochemical properties and different SPHK activities showed that the toxic effect was independent of SPHK inhibition (figure 4).

With a 100 fold separation between on-target SPHK inhibition and off-target cytotoxicity, compound A was selected for *in vivo* studies in mice. Compound A does not inhibit murine SPHK2 (table 1), and is specific for SPHK1 in murine cells. The effect of compound A on systemic S1P levels is similar to the SPHK1 knock-out mice, and leads to more than 70% reduction in plasma S1P levels (figure 7) [9]. Consistent with results reported with the S1P receptor modulator FTY720 and the S1P neutralizing antibody sphingomab, this reduction in extracellular S1P levels can influence parameters related to angiogenesis, in this case VEGF induced vascular permeability [22], [23]. The effects are not as strong as those observed with a VEGFR2 inhibitor, but reach statistical significance in female C57/B6 mice (figure 8), although not in athymic nude mice (data not shown). Systemic reduction of S1P levels does not lead to observable effects on cell viability in control or tumor bearing mice in the syngeneic B16F10 melanoma allograft model (data not shown), perhaps suggesting the S1P rheostat requires more complete SPHK inhibition to have observable effects on cell viability. However, this requirement was assessed in a xenograft model with the human breast cancer cell line MDA-MB-231. In this cell line, compound A can potently inhibit the intracellular S1P production (figure 2C). In addition, this model has been shown to be sensitive to S1P neutralization with sphingomab, with evidence pointing towards angiogenesis as a major mechanism of action [23]. The rheostat theory would predict reduced tumor cell viability under these conditions, but vehicle and compound A treated tumors grow at the same rate (figure 9).

In order to assess SPHK mediated effects on cell viability that are independent of SPHK’s enzymatic activity, large scale siRNA experiments were performed in more than 80 cell lines. Reduction of SPHK1 or SPHK2 expression did not lead to statistically significant effects on cell viability in any of these cell lines (figure 10). When siRNA targeting both SPHK1 and SPHK2 were combined in a small scale experiment, no increase in effect on cell viability was observable, but given the limitations of siRNA technology (incomplete knockdown, off target effects), those findings are not conclusive (data not shown).While these results are consistent with the observations obtained with compound A and compound B, it cannot be ruled out that potential effects caused by knockdown of one SPHK were compensated by the other isoform. It is also conceivable that none of the 20 SPHK targeting siRNAs achieved a level of knockdown necessary to cause a phenotype, although the knockdown of essential genes like PLK1 or POLR2A lead to readily detectable reductions in cell viability in this experimental setup (figure 11).

Taken together, the experiments presented here fail to produce any experimental evidence supporting the rheostat theory. These results are supported by a recent publication of the potent and specific SPHK1 inhibitor PF-543 [22], but contradict several earlier reports. These reports were either generated using imperfect tools like SKII or SK1-I, or looked at the effects of SPHK protein depletion with siRNA or shRNAs [2], [5], [6], [11]. It is conceivable that sphingosine kinases have functions independent of their kinase activity, which could explain some of these discrepancies.

The results presented here do not rule out that the S1P rheostat has a function under specific conditions not assessed in this study, or following SPHK activity ablation to a degree not achievable with SPHK inhibitors. Nevertheless, attempts to establish the intracellular S1P/sphingosine/ceramide axis as a major contributor to apoptosis and tumor cell viability *in vitro* and *in vivo* failed. In contrast to the role of S1P and its receptors in inflammation and angiogenesis, this study does not establish sphingosine kinases as promising drug targets for innovative cancer therapies.
